# Methods for Determining the Thermal Transfer in Phase-Changing Materials (PCMs)

**DOI:** 10.3390/polym12020467

**Published:** 2020-02-18

**Authors:** Vasile Bendic, Dan Dobrotă, Ionel Simion, Emilia Bălan, Nicoleta-Elisabeta Pascu, Dana Iuliana Tilina

**Affiliations:** 1 Faculty of Engineering and Management of Technological Systems, Politehnica University of Bucharest, 060042 Bucharest, Romania; vasile.bendic@upb.ro (V.B.); nicoleta.pascu@upb.ro (N.-E.P.); dana.tilina@upb.ro (D.I.T.); 2 Faculty of Engineering, Lucian Blaga University of Sibiu, 550024 Sibiu, Romania; 3 Faculty of Aerospace Engineering, Politehnica University of Bucharest, 060042 Bucharest, Romania; ionel.simion@upb.ro; 4 Faculty of Industrial Engineering and Robotics, Politehnica University of Bucharest, 060042 Bucharest, Romania; emilia.balan@upb.ro

**Keywords:** PCM, butyl stearate, polymethyl methacrilat, FEM, thermal transfer measurement

## Abstract

A very important issue that needs to be solved as simply and correctly as possible is how to establish the thermal performance of phase-changing materials (PCM). The undertaken researches have analyzed the values of the thermal performances of the PCM taking into account the method of finite elements and the experimental research, respectively, based on a modern measurement system that was designed and implemented. Butyl stearate which has been encapsulated through complex coacervation in polymethyl methacrylate has been used as a PCM. Samples were made containing 10%, 20%, 30% and 40% PCM, respectively, within their structure. The research has established that at both the hot plate and the cold plate interface, the evolution of the temperature over time, established by both the finite element method (FEM) and experimental research, are quite close, and the best results have been obtained for the P30 sample. A very important thing observed during the finite element method (FEM) is that the simulated thermal flow variation extends between 2700-3110W/m^2^ being small enough not to influence the temperature measurement at the interface of hot or cold plates. Thus, the use of the FEM or the experimental research method can be applied with good results, provided that the correct initial conditions are used in the finite element method and that the experimental research is performed using the best possible apparatus.

## 1. Introduction

The use of PCM materials ensures a reduction of thermal fluctuations inside buildings through the storage of heat in the form of a phase change. The use of these materials offers a maximum benefit when applied to buildings insulated with lightweight material as they have small intrinsic storage capacity. Thus, in order to achieve isolation with light energy efficiency, the following conditions must be met: reduced external energy exchange ensured by thermal insulation; the use of renewable energy sources; the reduction of energy requirements by using phase-changing materials [1,2,3,4,5].

For modeling thermal transfer in the case of PCM several parameters must be considered which can be divided into the following categories:

- Parameters related to the encapsulation geometry of PCM material and storage tank: the shape and size of the PCM capsule, the length, and diameter of the storage tank, the geometry of the capsules and storage tank;
- Parameters for fluid flow: flow, speed, properties of the fluid;
- parameters related to the response of the storage system such as: the initial state, inlet temperature, physical and thermal properties of the PCM, convection heat transfer coefficient, etc. [6,7,8,9].

In order to simplify the modeling of the thermal transfer in materials containing PCM, it is necessary to take into consideration an ideal passive solar precinct with interior PCM for storing energy, subject to cyclical indoor climatic conditions and internal heat sources. The assumptions to be taken into account in the modeling process are the following [10,11,12,13,14,15]:

- The sensitive thermal capacity of the package is slightly ignored;
- a single coefficient within, representing the sum of the thermal transfer by radiation and convection between the wall surfaces and the surroundings;
- ideal PCM panels have sufficient latent heat capacity at a single phase change temperature tm;
- as the thermal resistance 1/hin from the surface is high enough in comparison with the internal resistance of the thermal conductor of the thin PCM panel, the internal distribution temperature is assumed to be uniform;
- the surface of the PCM panel away from the room is assumed to be insulated (it is the case related to the inside cover of the building).

On undertaking the mathematical analysis of PCM composite materials, they are considered to be of a matrix type with spherical particles. In many cases, an idealized model is also envisaged, which implies an isotropic matrix with constant heat transfer coefficients, density and caloric capacity. Particles embedded in the matrix are also considered to have constant heat transfer coefficients, density and caloric capacity. The derivation of the mathematical model involves defining conductivity at the interface. The contact surface and conductivity at this interface is what separates the spherical particle from the surrounding matrix [16,17].

If the modeling is performed for PCMs of the type of butyl stearate that has been encapsulated in polymethyl methacrylate (PMMA) the problems related to the modeling and measurement of thermal transfer are much more complex as there is not only a lack of homogeneity of the PCM but also the presence of more material in the structure of the insulation material. In certain cases, thermal-cut modeling was achieved by the wire-to-face definition and application of Fourier law for modeling the normal heat-flow component along with the interface so that it should be proportional to the temperature difference between the two environments [18,19,20].

A possible solution for modeling the thermal transfer could be the use of the finite element method (FEM). This is based on the fact that numerical modeling with finite elements is one of the latest and most efficient methods of science and technology computing. However, the final element method is operative only if the algorithms built for its application are completed in a computer ‘understood’ version, in the shape of computer programs. Matrix-based techniques are used in the execution of these programs, and methods for solving large algebraic equation systems characterized by sparsely populated band matrix are developed [21,22,23,24,25].

There are numerous programs for analysis using the finite elements method: SAP 05, COSMOS, COMSOL, ANSYS, NASTRAN, PATRAN, IMAGE-3D, NOSAP, etc., some of them being specialized in solving a certain type of problem, others having a more general character. Under these circumstances, it is necessary to identify the best modeling program using FEM that allows the thermal transfer to be modeled for a PCM of the butyl acetate type that is encapsulated in polymethyl methacrylate (PMMA) [1].

An important issue that arises in relation to PCM refers to the construction of measuring equipment where the time constant of the test-device measurement system depends neither on the response time of the control system, the thermal capacity of the test piece, nor the components of the device in contact with it either [26,27]. One way to estimate the time constant is to initiate a step of change in the surface temperature of the hot plate, measuring the time required for this change to result in a heat flow measured on model 1/e from the total heat flow change, where the basis of the natural logarithm is to be found. In some measurements (especially in situ) data may vary over time in an apparently irregular manner. However, if there are no monotonous trends, this can be referred to as ‘quasi-equilibrium’ and the average of the variations can be calculated [28,29].

Under these circumstances, the research carried out aimed to establish the thermal performance for a PCM-type material made of butyl stearate and complex coacervated with polymethyl methacrylate by experimental research and FEM. Thus, the following topics were addressed in the work: the characterization of the material of the type PCM; the design of the heat transfer measurement system of the PCM; the modeling of the thermal transfer through FEM, the using of the Comsol Multiphysics 4.0a software; the thermal transfer simulation within the FEM measurement system; the analysis of the results obtained by modeling by FEM and experimental research.

## 2. Materials and Methods

### 2.1. Characterization of PCM Material

The PCM material used in the research has been butyl stearate, and it was used in the shape of microcapsules embedded by complex coacervation in a PMMA membrane (polymethyl methacrylate). The properties of butyl stearate are presented in Table 1.

The technological process for obtaining microencapsulated phase-transformation material (PCM) by complex coacervation relied on the fundamental principle of encapsulation, i.e., the coating of a substance (butyl stearate) with an inert polymethyl methacrylate (PMMA) membrane. On the basis of the process of obtaining microcapsules through complex coacervation, the mixing of two solutions of two hydrophilic colloids under appropriate conditions was taken into account. The action between the positive and negative loading of two polymers with pH change was also examined in the technological process of complex coacervation. Thus, after making the butyl stearate – water emulsion, the monomer has been attached to the surface of the dispersed phase with the aid of the surfactant. Azodiisobutyronitrile (AZDN) polymerization initiator is added during the last stage of microencapsulation for the development of the PMMA membrane.

Out of the multitude of phase-changing materials PCM, butyl stearate offers the best requirements for obtaining phase-transformation materials with higher characteristics due to the following characteristics: presenting a melting/solidification temperature of 19 °C; its low cost and availability; a large amount of energy stored at the phase change.

For the butyl stearate to be used under optimum conditions as a phase-changing material, it was necessary to develop a method of encapsulation so as to avoid leaks during phase changes. Thus, its retention was achieved through a micro-encapsulation process by complex coacervation within a PMMA membrane. The PMMA for the encapsulation of a butyl stearate has been chosen considering its physical properties (Table 2).

Butyl stearate, MMA and allyl-MMA used in the process of obtaining phase transformation material were purchased from Sigma Aldrich. (Saint Louis, MO, USA). As regards the azo- bisisobutyronitrile (AZDN), used as a polymerization initiator, it was purchased from ARKEMA Inc. (King of Prussia, PA, USA) and the surfactant Pluronic PE6200 was purchased from BASF (Houston, TX, USA). Iron sulphate (FeSO_4_·7H_2_O), ammonium persulphate and sodium thiosulphate (NaS_2_O_7_), used in the various stages of emulsifying, they were purchased from S.C. Alfa Vega SRL (Satu-Mare, Romania).

For the manufacture of the microcapsules of butyl/PMMA several stages of emulsification have been completed (Table 3) using a mini-emulsion device. 

In order to obtain the samples, during the first stage, a volume of 120 mL of deionized water was mixed within a mini-emulsion device to which a mass of 30 g of butyl stearate and 3 g of Pluronic PE6200 (surfactant) was added. As the emulsifying process, during the first stage, must be carried out at a temperature above 19 °C (the melting temperature of the butyl stearate), a working temperature of 30 °C has been selected. In order to establish the optimum emulsification technology, three methods were used, in different stages (Table 3).

### 2.2. Design of the Thermal Transfer Measurement System of the PCM

**Figure 1:**
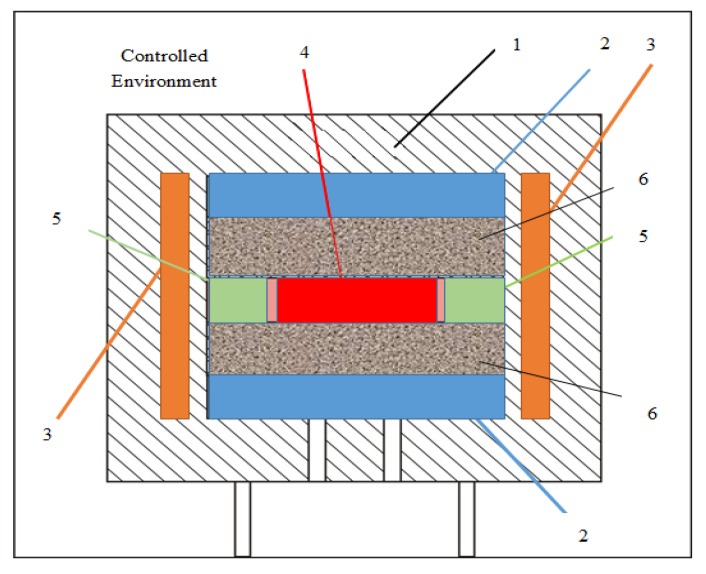
Structure of the test apparatus used. 1—insulation; 2—cold surface; 3—main thermal protection; 4—hot plate; 5—secondary thermal protection; 6—samples.

For measuring the conductivity of the phase transforming material (PCM) a testing method and apparatus were used, conforming to the ASTM C177 standard. The standard is intended for measuring stationary heat flow through flat and homogeneous samples, whose surfaces are kept in direct contact with the constant temperature plates. The test device constructed by this method is called bilateral apparatus with a protected hot plate. Figure 1 shows the main components of the test system used: two cold assemblies with isothermal surfaces and a hot assembly consisting of a hot plate representing the heat source and the thermal protection on the side. The general building parameters of the test apparatus are shown in Table 4.

The measurement of thermal conductivity with such a system produces a result which is the average of the two samples of material, therefore, it is important that the two samples should be identical. The calculation of the heat transfer coefficient has been carried out taking into account the C 1043, C 1044, C 1045 standards.

The hot plate-protective plate assembly is intended to produce an isothermic surface with a measurable heat flow (heat flow per unit of time) and defines the actual volume of the sample. The primary function of the protective plate is to provide suitable thermal conditions in the sample volume by reducing the lateral heat flow within the sample. The operation of the appliance requires a heated tank, which is secured by hot plates. A multi-layered structure of copper, aluminum oxide and aluminum have been chosen for the practical application of this plate.

Electrical resistors have been produced by the chemical erosion of a pre-fabricated copper-aluminum-oxide-aluminum laminate. A photo-resistant material has been applied to the copper surface in order to obtain the trace. The photo-resistant material was exposed to a UV light source together with the film representing the geometry of resistance. After chemical corrosion, the electrical resistance routes 0.5 mm thick have been obtained with an electrical resistance of 25 to 28 Ω for the central resistance and 31 to 35 Ω for the protective resistors.

The following computation relationship has been used to determine the resistance of the heating element:

(1) $$R_{\left(T\right)}=\frac{\rho_{\left(T\right)}\times l}{a}$$

where: $R_{\left(T\right)}$ is the resistance of the conductor [Ω], $\rho_{\left(T\right)}$ the specific resistance of the material [Ω·m], *l* the length of the conductor [m]; *a* the area of the conductor cross section [m^2^]:

(2) $$\rho_{\left(T\right)}=\rho_{0}\times \left[1+\alpha \left(T-T_{ref}\right)\right]$$

where: $\rho_{0\;}$ is the resistivity of the material to $T_{ref}$; the variation coefficient of resistance to temperature; *T*—the temperature of the material; *T_ref_*—the reference temperature.

Two RXN-303D laboratory power sources with programmable power and intensity were used to tow electrical resistance in the intervals: 0–30 V, 0–3 A. Due to the use of a programmable source, the total power could be determined and therefore the energy input into the system according to the equation:

(3) Q=V·A [W]

A NTC model EC95F103V produced by GE SENSING (Billerica, MA, USA) with a tolerance of ±0.1 °C has been used to measure the temperature within the measuring apparatus.

**Figure 2:**
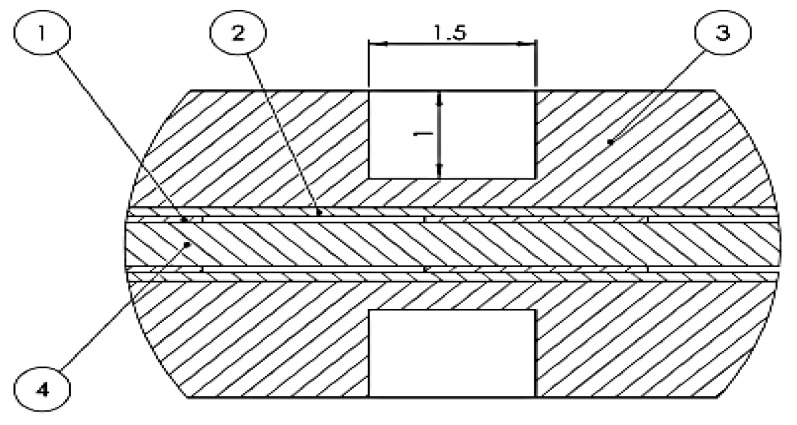
The hot plate constructive elements. 1—electrical resistance made from Cu; 2—dielectric layer made of AlO, 3—conductive layer made of Al, 4—dielectric separationlayer.

**Figure 3:**
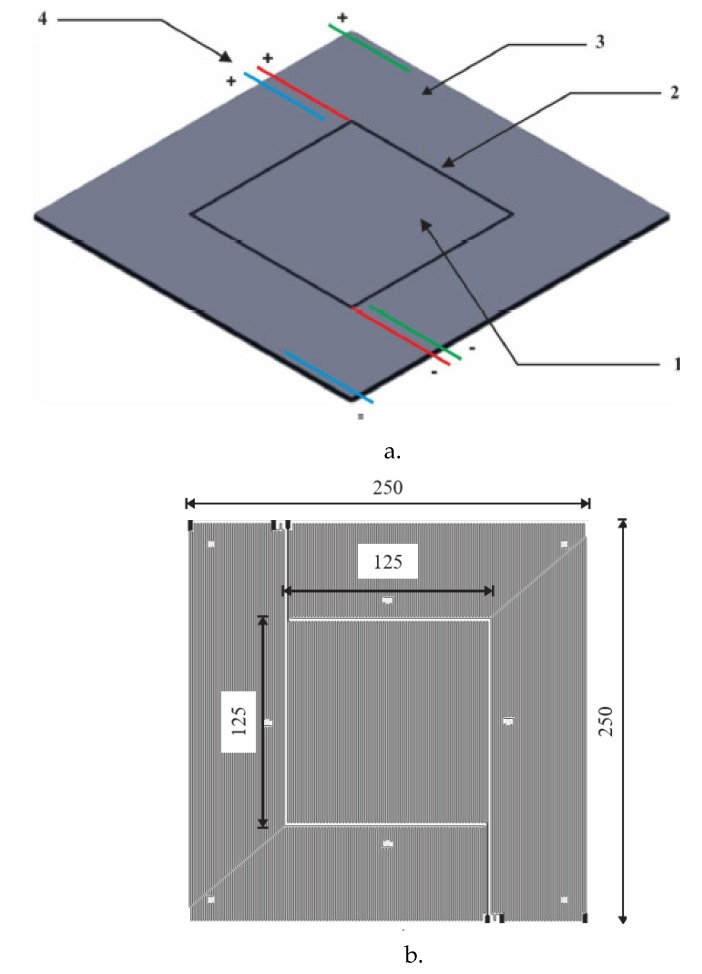
Hot plate assembly. (**a**)—structure of the hot plate; (**b**)—effective geometry of the path of the electric resistors; 1—main resistence; 2—protection slot; 3—protection resistance; 4—electrical connections.

The hot plate is characterized by a series of elements (Figure 2). The hot plate assembly is shown in Figure 3.

**Figure 4:**
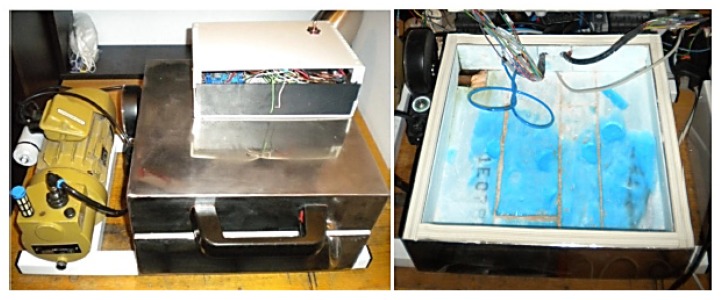
The stand used in experimental research.

The designed and realized experimental stand (Figure 4), consists of a measuring precinct, a vacuum pump, an electronic module, and two programmable power sources. The measuring precinct sizing 90 mm × 350 mm × 350 mm was made of 308L stainless steel with a panel wall thickness of 2 mm. It has an insulating wall with a thickness of 40 mm inside it.

In order to minimize errors due to the heat losses of the sample by convection, it has been chosen that measurements should be made under vacuum. The pressure used during the experiments amounted to 10 torrs, the precinct being capable of maintaining this pressure for approximately 3-4 h without the vacuum pump intervention. Maintaining low pressure was possible by using a suitable insulation gasket and an electrovalve.

The electronic module of the measuring device was made of a data acquisition and control board, voltage divider plate and vacuum pump control relay. The LabJack U3 board model, manufactured by LabJack Co. (Lakewood, CO, USA), has 16 analog inputs and two analog outputs. Out of the 16 analog inputs, 14 were assigned to temperature sensors, an analog input was used to measure the pressure in the measuring precinct and another analog input was used to measure the voltage input into the voltage divider plate.

**Figure 5:**
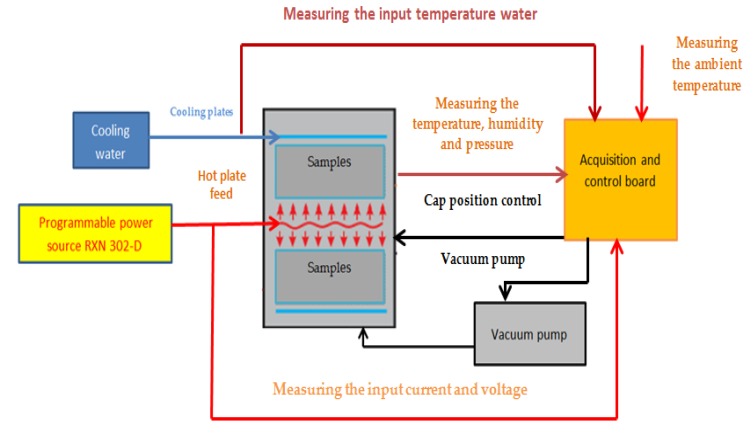
The logical operating diagram of the stand used in experimental researches.

The two analog outputs were assigned to supply the thermistor and control the vacuum pump. The supply of the hot plate has been done using two programmable sources, models RXN-302D and RXN-303D (ITEC Ltd., Tel Aviv, Israel), capable of providing a maximum voltage of 30 V and a maximum power of 2 A and 3 A, respectively. The RXN-302D source was used to supply the central resistor and the RXN-303D source to supply the protection resistors. The logical layout of the stand used in the experimental researches is shown in Figure 5.

### 2.3. Modeling Thermal Transfer through FEM Using the Comsol Multiphysics 4.0a Software

COMSOL Multiphysics 4.0a (COMSOL Multiphysics^®^ software, Los Angeles, CA, USA) was the finite element program used in the experimental studies. The elaboration of a fair and efficient model of a real PCM material requires a synthetic schematization of that material. A thorough analysis of the real phenomenon was necessary to properly shape it, through an in-depth knowledge of it, as well as through the removal of the insignificant aspects for its intended purpose, by making a synthesis as simple as possible.

**Figure 6:**
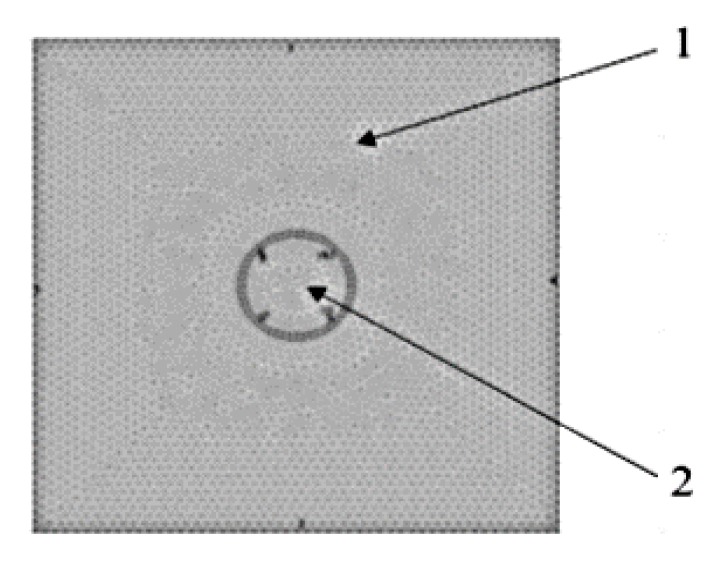
Discretization with FEM method of intelligent composite: 1—matrix composite material; 2—the armature.

The envisaged simulated design was represented by the matrix phase within which a PCM micro-capsule was embedded The simulation program has a vast database containing the properties of materials, so the materials chosen for the matrix and for micro-capsule coating were portland cement for the matrix and PMMA for the encapsulation material. A temperature difference of 20 °C at two of the material boundaries has been established as input data for achieving the simulation. The simulation results are shown in Figure 6 present the discretization with finite elements (finite element network).

## 3. Results and Discussion

**Figure 7:**
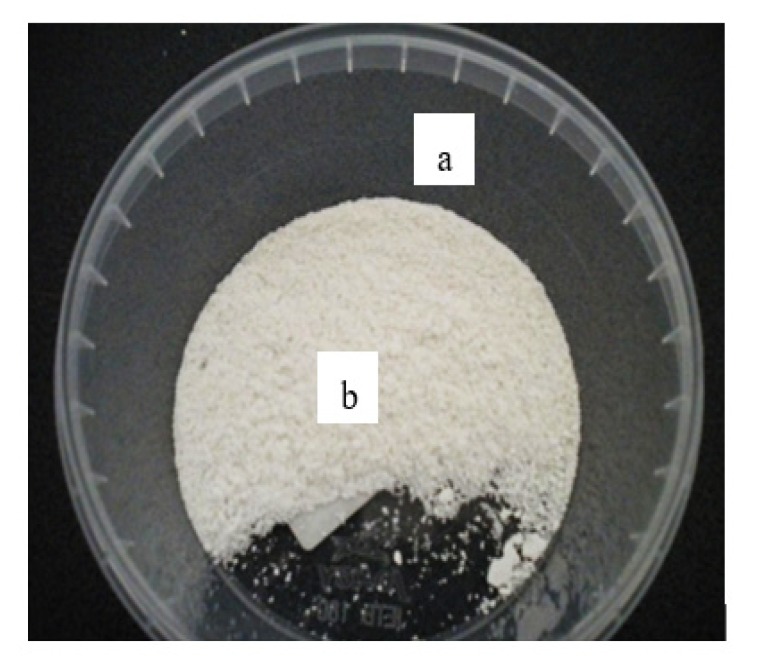
Butyl stearate microcapsules encapsulated in a PMMA membrane—PCM I. (**a**) storage bowl; (**b**) microcapsules.

Experimental researches started with the elaboration of butyl stearate microcapsules encapsulated in a PMMA membrane. Thus, following all the emulsification steps, a white precipitate was obtained that was subject to a relaxation process for 45 min. After the relaxation process, the precipitate had to be separated from water and this operation was performed under vacuum for 24 h, at 20 °C. The resultant butyl stearate microcapsules encapsulated in the PMMA membrane are shown in Figure 7.

**Figure 8:**
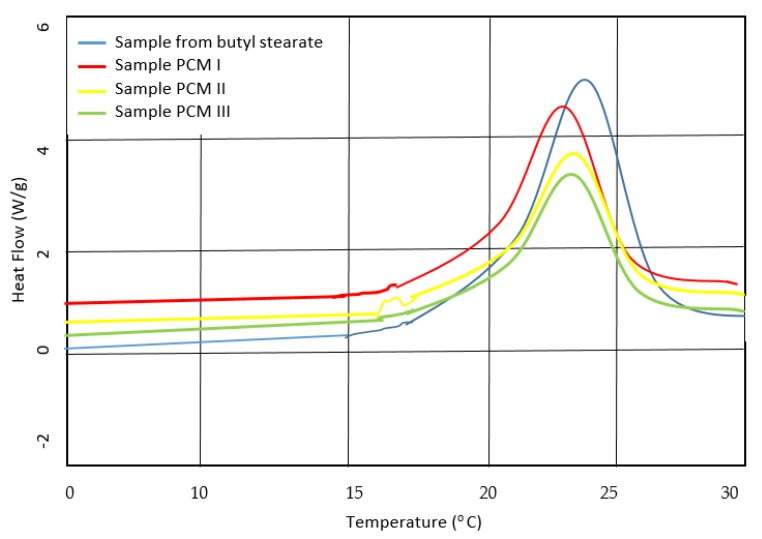
DSC diagram presenting testing results.

For the analysis of the thermal behavior of the obtained samples by the three emulsification technologies, but also of the butyl stearate, and the comparison of the results, a DSC differential calorimeter (Q20, TA Instruments-Waters LLC, Giessen, Germany) was used which allows the temperature adjustment from −90 to 450 °C with an adjustable accuracy of ±0.1 °C, respectively of the enthalpy of ± 0.1%. The results of these comparisons are shown in Figure 8.

Comparative analysis of the thermographs shown in Figure 1 indicated, as expected, that the sample of pure butyl stearate has the best heat storage properties. This can be explained by the fact that the other three samples have a lower thermal energy absorption capacity, due to coacervation in a PMMA membrane. However, the presence of the PMMA membrane confers a significantly higher physical stability, compared to the classical sample made only of butyl stearate. Also, for the other 3 samples, obtained according to the technologies presented in Table 3, the best heat storage properties had the sample I. In these conditions, in the subsequent researches, microcapsules made with the first emulsification technology were considered, which was presented in Table 3 (PCM I).

**Figure 9:**
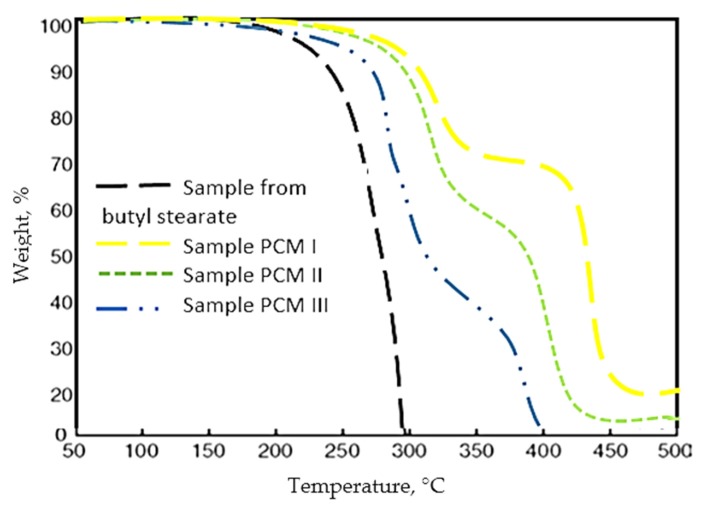
TGA curves of butyl stearate and sample PCM I, PCM II, PCM III.

Thermogravimetric analysis (TGA) of microcapsules was performed using a TGA/DSC1 instrument equipped with STAR software (Mettler Toledo, Giessen, Germany). TGA analysis was performed for both butyl stearate and to the microcapsules obtained by different emulsification techniques (Figure 9).

From the TGA analysis, it was observed that the mass loss is much higher and at a much lower temperature in the case of the butyl stearate sample. In the case of PCM I, PCM II, PCM II, the thermogravimetric analysis showed that they have a very good thermal stability compared to PCM made only from butyl stearate. Thus, precisely the micro-encapsulation of PCM in PMMA prevents mass loss of the samples, and the best behavior had PCM I sample.

**Figure 10:**
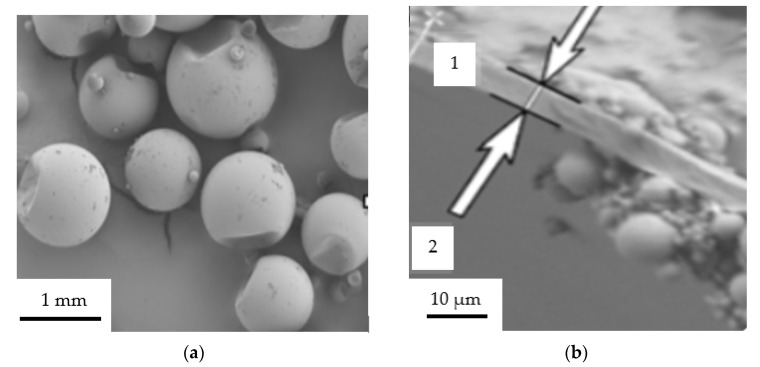
Images obtained by electron microscopy of PMMA-butyl stearate microcapsules—PCM I. (**a**)—microcapsules image—PMC I; (**b**)—microcapsule section (1—butyl sterate; 2—membrane of PMMA).

Also, a Scanning Electron Microscopy (SEM) analysis of PMC I was performed (Figure 10). A SU3800/SU3900 scanning electron microscope (Hitachi High-Technologies, Krefeld, Germany) was used for SEM analysis.

The microcapsules obtained were characterized by a high friability and had dimensions between 0.5–3.5 mm. The butyl stearate microcapsules encapsulated in a PMMA membrane had a fairly uniform particle size distribution. Also, the co-preservation process was an appropriate one in that the PMMA layer was relatively uniform in thickness, and this was observed after electron microscopy analysis (Figure 10). Also, considering the amount of PMMA butyl stearate used in the co-preservation process, but also how PMMA membranes formed around butyl stearate, an encapsulation ratio of 37.5% was established (the percentage of butyl ester from a PCM I capsule).

Regarding the obtained microcapsules, they were also characterized by the point of view of polydispersity using the optical microscopy, and the obtained values were processed using analysis of variance (ANOVA). Thus, the estimation of the average size of the microcapsules (numerical mean size *D_n_*, mean gravimetric size *D_w_*) and the polydispersity index (*D_w_/D_n_*) was made by measuring 100 microcapsules:

(4) $$D_{n}=\frac{\sum_{i}N_{i}\cdot D_{i}}{\sum_{i}N_{i}}$$

(5) $$\;D_{w}=\frac{\sum_{i}N_{i}\cdot D_{i}^{4}}{\sum_{i}N_{i}D_{i}^{3}}$$

(6) $$PDI=\frac{D_{w}}{D_{n}}$$

where: *Ni* represents the number of particles that have the size *Di*.

After measuring the dimensions of the microcapsules and calculating the numerical average size Dn, respectively the gravimetric mean size Dw, the value of the polydispersity index PDI = 0.729 was determined.

The developed composite material consisted of butyl stearate microcapsules encapsulated in a PMMA membrane, AlO representing the reinforcement phase and cement representing the matrix phase. As for the specimens, they were made considering different proportions of the mass of microcapsules of type PCM I. Thus, four specimens were made which had the following proportions of PCM I: 10% PCM (P10), 20% PCM (P20), 30% PCM (P30), 40% PCM (P40). The composition of the tested specimens is presented in Table 5.

All the ingredients in the composition of the samples were mixed using an electrically operated mechanical mixer (Dinc Makina, Istanbul, Turkey). First, the required quantities of cement were mixed with a quantity of water (water/cement ratio was selected as 0.33) and AlO. Finally, PCM I was added and mixed for 5 min. After that, the samples were immediately poured into the molds. After 1 day, the concrete specimens were removed from the molds and cured in water at a temperature of 20 ± 2 °C until the age of 7 and 28 days. In order to obtain an optimal curing process, specimens were fully submerged in water baths throughout the process.

**Figure 11:**
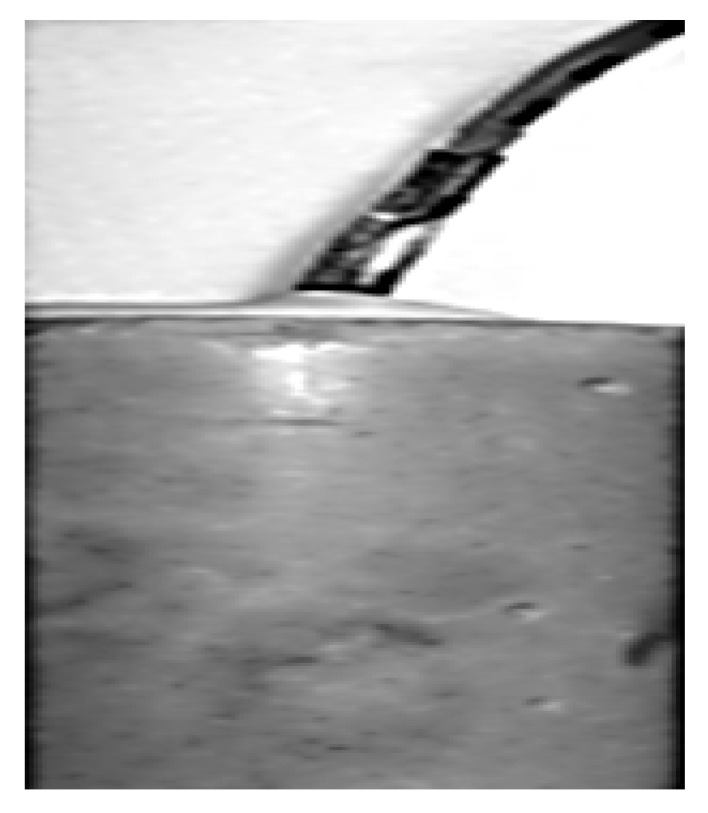
Test specimens used.

The test-pieces were prismatic and have sizes to 250 × 250 × 50 mm^3^ (Figure 11). After moulding the samples, a temperature sensor was inserted into the center of each test-piece, using a Ø 6mm bore hole, the vacant space being subsequently filled with cement.

The mixing report made was the following: matrix (cement) in a proportion of 50%–80% of the total mass; AlO in the percentage of 10%; PCM I microcapsules of 10%–40%. The samples had different masses depending on the content of PCM I. Thus, the individual masses of the obtained samples and the masses of the three materials in their structure are presented in Table 6.

**Figure 12:**
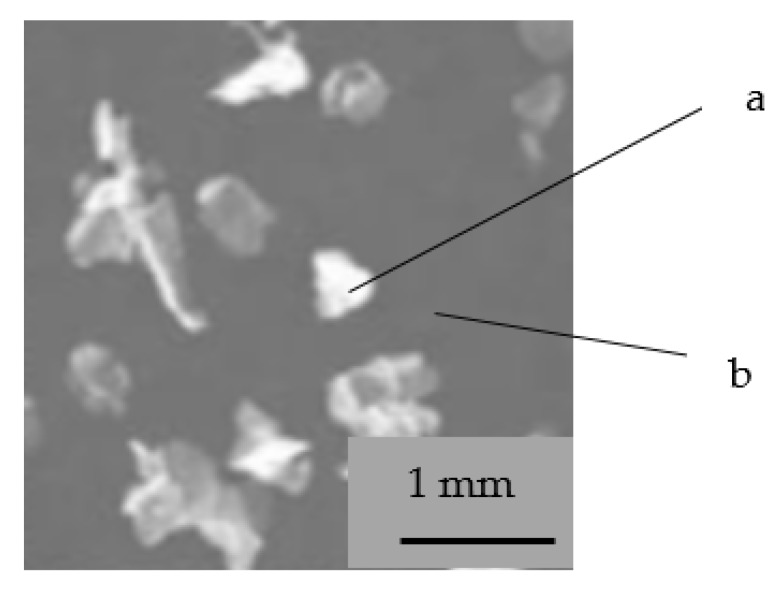
Section through P30 sample **a**—microcapsules of PCM I, **b**—cement matrix.

In order to ensure that the measurements made are not accompanied by errors during the first stage, the test specimens have stabilized and thus undergone 50 melting-solidification cycles. To achieve this, a model 9106 thermostated bath produced by PolyScience has been used. The bath is capable of reproducing temperatures in the range of −20 ÷ +150 °C. For the experiments carried out, the temperatures of 2 °C have been used as the solidification temperature and 25 °C as the melting temperature of the PCM material. Also, a section through the P30 sample was made and an image of obtained through the optical microscopy is shown in Figure 12.

### 3.1. Thermal Transfer Simulation within the FEM Measurement System

**Figure 13:**
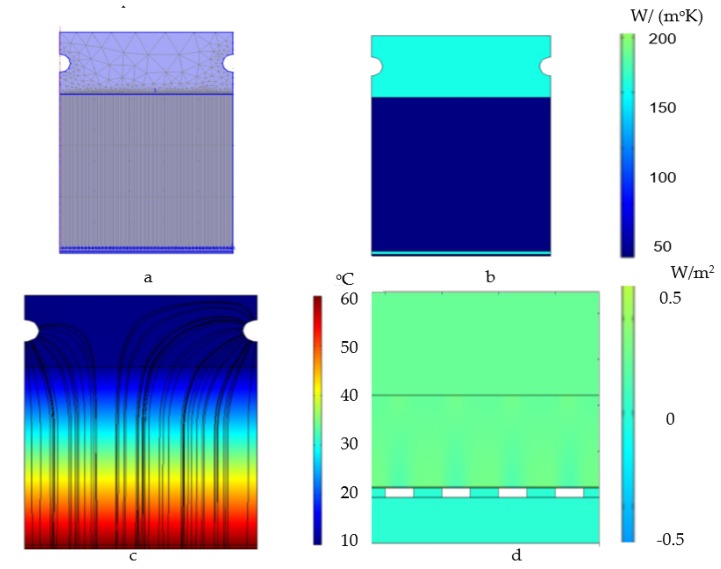
Simulation of the thermal transfer within the measurement system by the FEM method: (**a**)—representation of the geometry and mesh achieved; (**b**)—the thermal conductivity, in W/(m·K) of the simulation elements; (**c**)—thermal flow (continuous lines) and temperature through section (**d**)—detail on thermal flow, in (W/m^2^) in the area of the hot plate.

In order to observe the phenomenon of thermal transfer within the measuring device, numerical simulation was used with the help of Comsol Multiphysics 4a. The simulation was carried out in 2D-axis-symmetrical mode, where a section through the center of the measurement system was chosen as the symmetry plane. The measuring system was represented by an assembly of the hot plate—measured material—cold plate, so the measured material is inferior bounded to the hot “tank” and superior to the cold “tank”. The used mesh is shown in Figure 13a, and is made of 5764 elements with a total surface of 5551 mm^2^.

The geometry used in the simulation was made on the basis of the real one, consisting of: a small dielectric mica layer of 0.5 mm thickness; electric resistors made of copper 0.1 mm thick; a dielectric layer made of aluminum oxide, with a thickness of 0.1 mm; a support layer of aluminum, with a thickness of 1 mm; the measured material, with a thickness of 50 mm; an aluminum plate with a thickness of 20 mm.

The materials from which the simulated elements are made were also chosen on the basis of the actual construction of the thermal transfer measuring apparatus. Since the characteristics of the measured material were not known at the beginning of the experimental researches, its chosen thermal properties were those of the usual concrete. The temperature chosen for the cold plate has been 10 and 60 °C. for the hot plate. Figure 13b shows the thermal conductivity of the selected materials and Figure 13c shows the results of the simulation: the thermal flow (continuous lines) and the temperature through the simulated section.

In Figure 13d, the heat flow in the hot plate area from the electrical resistance to the test-piece can be observed. The numerical simulation indicated a heat flow variation of 0.7 W/m^2^ at the interface between the electrical resistance and the conductor layer made of Al. However, the variation of the thermal flow becomes uniform up to the interface between the conductor layer of Al and the surface of the test-piece, thus resulting in a uniform temperature at the interface.

**Figure 14:**
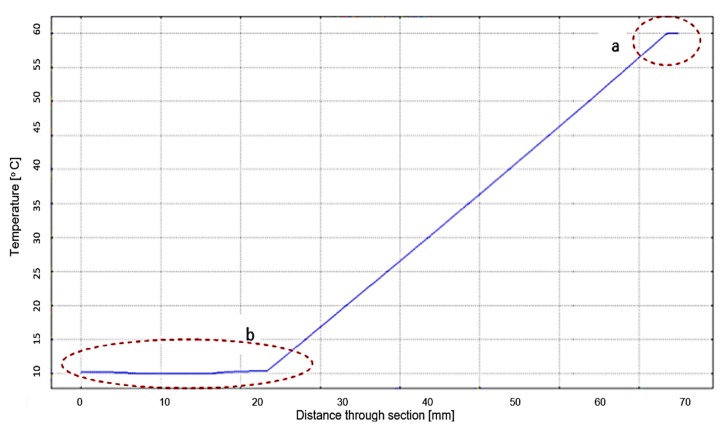
Temperature variation depending on distance trough section: (**a**)—temperature at the hot plate interface; (**b**)—temperature at the interface and through the cold plate section.

Figure 14 shows the graph of the temperature change through the simulated section. Notice is to be made of the temperature uniformity at the hot plate interface (Figure 14a). A deviation of 1 °C from the cooling water temperature of 10 °C can be observed at the cold plate interface (Figure 14b), indicating possible under-sizing of the cooling plate. However, this deviation is small enough not to influence the operation of the measuring device.

**Figure 15:**
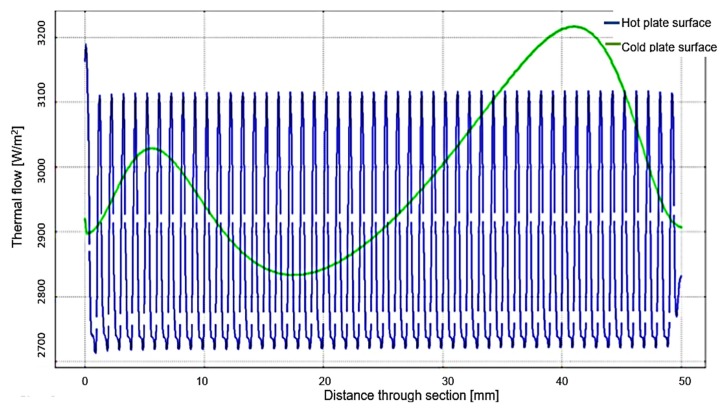
Variation of thermal flow at the contact surface between the measured material and the two plates in W/m^2^.

Following the variation of the thermal flow at the contact surface between the measured material and the two plates shown in Figure 15, an irregularity is observed at the interface of the material with the hot plate. The simulated thermal flux variation is between 2700–3110 W/m^2^ and has a sinusoidal shape. The period of sinusoidal variation is small enough not to influence the temperature measurement at this interface.

### 3.2. The Analysis of the Results Obtained by Modeling Using FEM Respectively the Experimental Research

In order to analyze the correspondence between the results obtained in the modeling process using the FEM method and those obtained via the experimental research, the 4 samples (P10, P20, P30, P40) have been tested. The temperature was measured at the interface of the hot plate, respectively the interface of the cold plate. To measure the temperature at the two interfaces, they used NTC thermistors model EC95F103V produced by GE SENSING, with a tolerance of ± 0.1 °C. The results were recorded following a cyclic loading of the developed butyl stearate microcapsules.

In order to carry out the experimental researches with the help of the designed stand, an experimental procedure was developed consisting of the following stages:

− positioning of the temperature sensors at the interface between test-cold plate and test-hot plate;
− positioning of thermistors as follows: 3 in the area of the central metering resistance, the upper part, 3 in the area of the central metering resistance, the lower part, one in the area of the protection slot, the upper part; one in the area of the protection slot, the lower part; 3 in the protection zone, the lower part; 3 in the protective zone, the lower part;
− introduction of the assembly made in the measuring enclosure;
− connecting cold plates to the water network;
− starting the power sources and connecting the hot plate to them;
− adjusting the heating voltage/current of the central metering resistor;
− connecting to the PC the acquisition card and starting the data acquisition program;
− setting the operating range of the vacuum pump and starting it in the “automatic” mode of the program interface; initial temperature stabilization (2–3 h);
− adjusting the heating voltage/current of the protection resistance so as to obtain a maximum temperature difference of 0.4 °C between it and the central metering area;
− secondary temperature stabilization (4–6 h);
− data collection.

**Figure 16:**
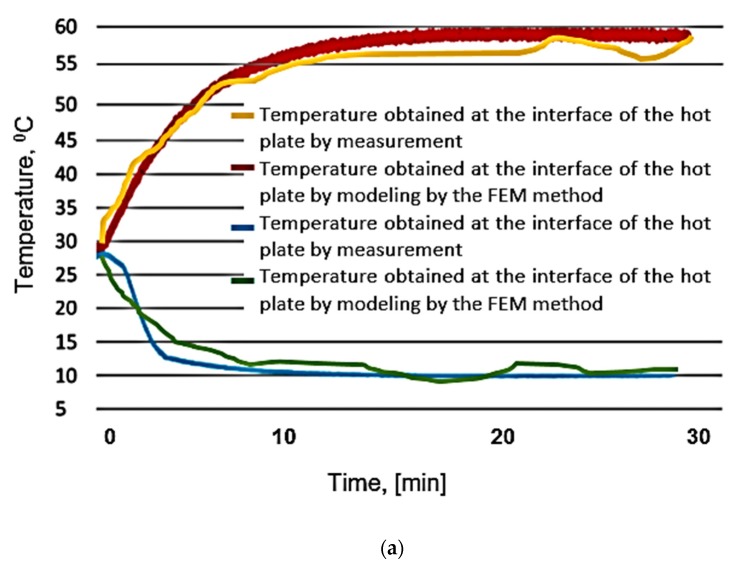

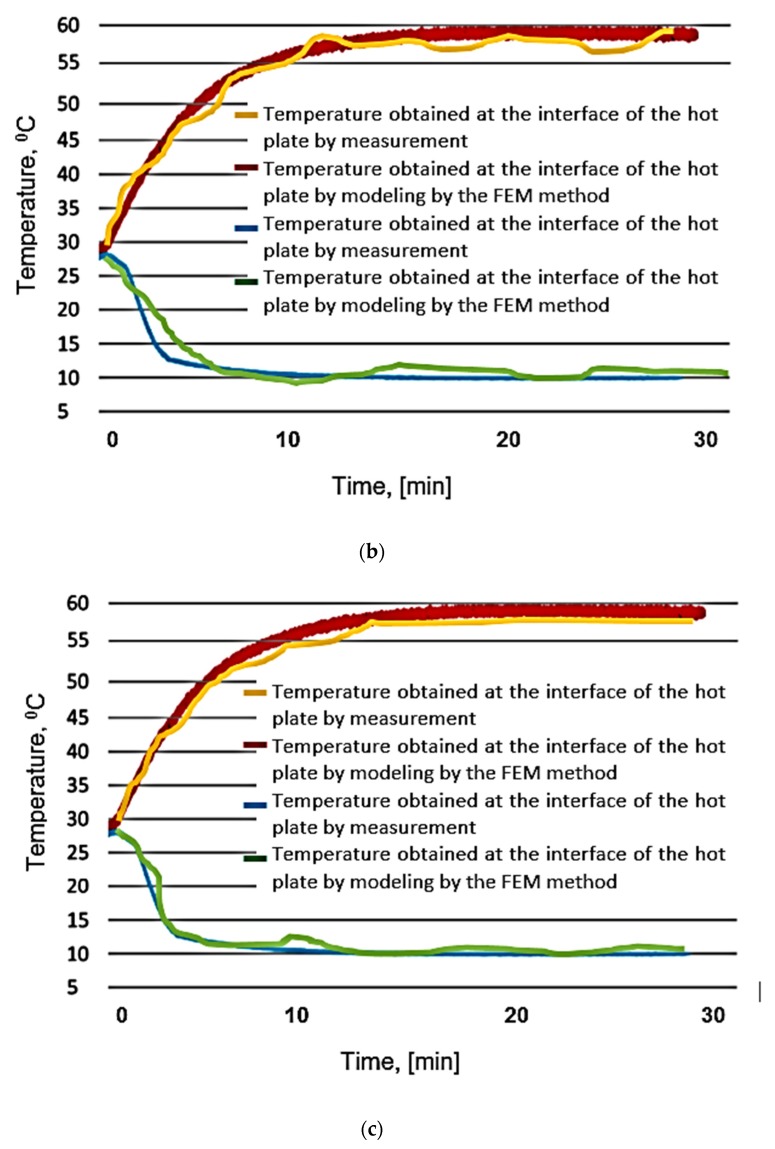

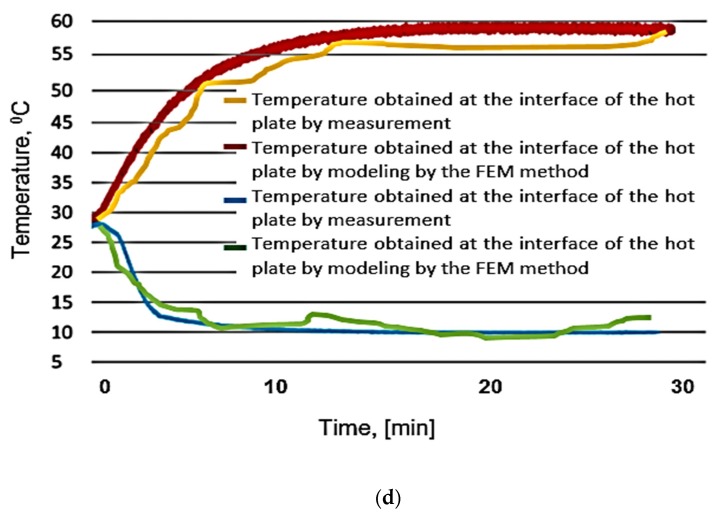
Temperature variation in time for test specimens: (**a**)—P10, (**b**)—P20, (**c**)—P30, (**d**)—P40.

The temperature at the hot and cold interfaces for the four types of samples has also been determined using the FEM method. The results obtained are shown in Figure 16.

The analysis of the temperature evolution in time presented in Figure 16, both at the hot plate interface and at the cold plate interface, revealed the fact that the results obtained through the FEM model are close to those obtained through the experimental measurements. The best results have been obtained for P30 samples. This finding demonstrates that the optimal percentage of PCM microcapsules that have to be found in the structure of some building materials must be around 30%. A very important aspect observed during the modeling process by FEM is that the simulated thermal flow variation is between 2700–3110 W/m^2^, being small enough not to influence the temperature measurement at this interface of the hot or the cold plates. Due to the programmable source, the total power and therefore the energy on entering the system could be determined. A very important thing obtained from experiments carried out on intelligent composite materials refers to the establishment of temperature values at the interface of the two plates, taking into account thermal behavior, in the context of cyclic loading of the developed butyl stearate microcapsules.

Thus, in general, FEM simulations could be used to evaluate the range of the functioning conditions under which PCMs could be considered to behave as a homogeneous material. However, experimental validation is always required, depending on the specific heating conditions. Considering these conditions, a more extensive and carefully planned experimental testing is always required in order to obtain close results between FEM and the experimental research. Equipment having the constructive form presented in the paper can satisfy the best experimental research conditions and thus the conditions to control the heat flow or temperature on the hot/cold surface could be fulfilled.

The above simulations demonstrate the fact that the presence of the passive phase could affect the response to the temperature variation over time for PCMs. Thus, in general, FEM simulations could be used to evaluate the range of operating conditions under which PCMs might be considered to behave as a homogeneous material, but it needs experimental validation under specific heating conditions. Further experimental research is needed when the possibility of taking into account the usual structures or the area of the interface between the cement matrix and the PCMs is prevented. A wider range of experimental conditions could be realized in view of a new experimental setup that could provide information on the heat flow or temperature on the hot surface respectively the cold surface.

## 4. Conclusions

The analysis undertaken regarding the thermal transfer in the materials containing a PCM has shown that both at the interface of the hot plate and at the interface of the cold plate, the evolution of temperatures over time, established both by the finite element method (FEM) and by the experimental research, in both cases, are quite close to one another and the best results have been obtained for the P30 samples. A very important thing found during the modeling process by the finite element method (FEM) is that the simulated variation of the thermal flux is between 2700–3110 W/m^2^, being small enough not to influence the temperature measurement at the interface of the hot plate, respectively cold plate. Thus, the use of FEM or the experimental research method can be applied with good results, provided that, in the case of FEM, the correct initial conditions are imposed and the experimental research is performed using a high-performance device. Also, the DSC and TGA analyses allowed the optimum coacervation conditions to be obtained so as to obtain the best PCM with butyl stearate structure respectively a PMMA membrane.

The design of a complex experimental configuration aimed at characterizing the thermal behavior of PCMs can be an optimal solution that allows both an instantaneous analysis of the obtained results and the optimization of the PCMs structure. The experimental results suggest that the presence of the PMMA membrane to some extent impedes the thermal transfer, but gives the microcapsules very good resistance and stability. The presented experimental research can contribute to the establishment of the boundary conditions necessary for the experimental identification or validation of the operational intervals in which PCMs can be considered homogeneous materials with suitable thermophysical properties.

Future research will determine whether the methods used to characterize the performance of PCMs made of butyl stearate encapsulated by complex coacervation in polymethyl methacrylate can be applied with good results to other PCMs.

## Figures and Tables

**Table 1:** Properties of butyl stearate.

Properties	Values
Grade	Technical
Assay	40–60% (GC)
Refractive index	N20/D 1.443
Melting Point	17–22 °C
Flash Point	160 °C
Density	0.861 g mL^−1^ at 20 °C
Molecular weight	340.58 g mol^−1^

**Table 2:** Physical properties of polymethyl methacrilat, PMMA.

Temperature, °C	−200	−150	−100	−50	0	20	50	100	150	200
Caloric capacity, KJ/Kg °K	0.67	0.90	1.06	1.26	1.26	1.42	1.85	0.67	0.90	1.06
Thermal conductivity, W/m °K	0.16	0.18	0.19	0.19	0.19	0.19	0.20	0.19	0.18	0.16
Coefficient of linear thermal expansion, 1/^0^K	3.0	3.7	4.5	5.7	6.9	7.5	12.0	18.4	3.0	3.7

**Table 3:** Stages of sample emulsification technology.

Sample Number	Emulsifying Step	Components	Obs.
I	EM_1	H2O Surfactant Butyl stearate	-
	EM_2	EM_1 MMA FeSO4 Ammonium persulfate	-
	EM_3	Na2S2O7	-
	EM_4	AZDN	after 24 h of manufacture
II	EM_1	H_2_O Surfactant Butyl stearate	-
			rigorous stirring
			after 10 min of relaxation
	EM_2	EM_1 MMA FeSO4 Ammonium persulfate	-
	EM_3	Na_2_S_2_O_7_	-
	EM_4	AZDN	-
III	EM_1	• H_2_O • Surfactant • Butyl stearate	-
	EM_2	EM_1 MMA	after 24 h of manufacture
	EM_3	• FeSO4 [0.0015 g]	-
	EM_4	Ammonium persulfate	after 2 min of relaxation
	EM_5	• Na_2_S_2_O_7_	after 24 h of manufacture

**Table 4:** General constructive parameters of the test apparatus.

Construction Parameters	Condition Imposed	The Realised Value
Hot surface	Material with adequate heat transfer	Al
	Temperature deviation in the plan	0.2 °C<
	Emissivity	<0.8
	Flatness	0.025% < from the value of length
	Appropriate size	125 mm × 125 mm
Main thermal protection	Side section size = ½	62.5 mm
	Temperature deviation in the plan	0.2 °C<
	Emissivity	<0.8
Protective slot	Area ≤ 5% Active surface area	1.5 mm in the section

**Table 5:** Composition of tested samples, % wt.

Samples	PCM I	AlO	Cement
P10	10	10	80
P20	20	10	70
P30	30	10	60
P40	40	10	50

**Table 6:** Masses of the samples and of the materials in their structure, Kg.

Samples	Mass of the Samples	Mass of the Portland Cement	Mass of AlO	Mass of the Microcapsules of the PCM I (Stearate de Butyl + PMMA)	Mass of the Butyl Stearatre
P10	6.52	5.216	0.652	0.652	0.244
P20	6.09	4.263	0.609	1.218	0.456
P30	5.65	3.390	0.565	1.695	0.635
P40	5.31	2.655	0.531	2.124	0.796
